# Telemedicine in the Era of a Pandemic: Usefulness of a Novel Three-Lead ECG

**DOI:** 10.3390/diagnostics13152525

**Published:** 2023-07-28

**Authors:** Milos D. Babic, Stefan Veljkovic, Jovana Lakcevic, Rade Babic, Miodrag Ostojic, Masa Petrovic, Darko Boljevic, Stanko Tomic, Milovan Bojic, Aleksandra Nikolic

**Affiliations:** 1 Dedinje Cardiovascular Institute, 11000 Belgrade, Serbia; babicmisa@hotmail.com (M.D.B.); mostojic2011@gmail.com (M.O.); dedinje@ikvbd.com (M.B.);; 2 Faculty of Medicine, University of Belgrade, 11000 Belgrade, Serbia; 3 Vinca Institute of Nuclear Sciences,11000 Belgrade, Serbia; stanko.s.tomic@googlemail.com

**Keywords:** electrocardiogram, COVID-19, pandemic, telemedicine, medical devices, signal processing

## Abstract

The 12-lead electrocardiogram (ECG) is a first-line diagnostic tool for patients with cardiac symptoms. As observed during the COVID-19 pandemic, the ECG is essential to the initial patient evaluation. The novel KardioPal three-lead-based ECG reconstructive technology provides a potential alternative to a standard ECG, reducing the response time and cost of treatment and improving patient comfort. Our study aimed to evaluate the diagnostic accuracy of a reconstructed 12-lead ECG obtained by the KardioPal technology, comparing it with the standard 12-lead ECG, and to assess the feasibility and time required to obtain a reconstructed ECG in a real-life scenario. A prospective, nonrandomized, single-center, adjudicator-blinded trial was conducted on 102 patients during the COVID-19 pandemic at the Dedinje Cardiovascular Institute in Belgrade. The KardioPal system demonstrated a high feasibility rate (99%), with high specificity (96.3%), sensitivity (95.8%), and diagnostic accuracy (96.1%) for obtaining clinically relevant matching of reconstructed 12-lead compared to the standard 12-lead ECG recording. This novel technology provided a significant reduction in ECG acquisition time and the need for personnel and space for obtaining ECG recordings, thereby reducing the risk of viral transmission and the burden on an already overwhelmed healthcare system such as the one experienced during the COVID-19 pandemic.

## 1. Introduction

The coronavirus (COVID-19) pandemic rapidly transformed the daily lives of patients and healthcare providers worldwide. To address the issues faced within healthcare during the pandemic, the leading professional associations such as the American Association of Cardiologists, the World Health Organization (WHO), as well as other international and local public health organizations put a tremendous effort into actively updating recommendations and suggestions calling for the broader use of telemedicine. Telemedicine has been recognized as one of the technologies having the ability to dramatically reduce the possibility of viral transmission, thus reducing the overall burden on healthcare worldwide [1,2]. Since the beginning of the pandemic, telemedicine has been demonstrated to be a feasible, acceptable, and effective healthcare modality in Western China, significantly improving healthcare outcomes [3].

It has been continuously shown that COVID-19 is linked with a multitude of cardiovascular complications, which most often include acute myocardial injury due to acute coronary syndrome (ACS), myocarditis, stress-cardiomyopathy, arrhythmias, cardiogenic shock, and cardiac arrest. It is important to recognize that these complications can also be present in asymptomatic patients [4]. Furthermore, the use of antiviral drugs could also cause an increase in the QTc interval for up to 72 h after treatment, with the prolongation of the QTc interval observed in 36.3% of patients [5]. Some studies have also suggested that a combined arrhythmogenic effect of the SARS-CoV-2 infection and hydroxychloroquine could account for the excess of QTc prolongation and fatal arrhythmias previously described in some COVID-19 patients [6]. 

The 12-lead ECG is the most widely used tool that aids in the early diagnosis of heart diseases. During the COVID-19 pandemic, with the restrictions in place and shift in work and daily living routines, there was an increased importance on monitoring cardiac manifestations and adverse therapeutic effects; however, this presented a challenge during the pandemic because the proper use of the standard 12-lead ECG requires trained and dedicated personnel, along with specialized equipment, which can only be performed at the patient’s bedside [7]. The shortage of healthcare workers during the pandemic and the time required to obtain a standard ECG ultimately led to a situation where ECG recording was unable to be performed in a routine daily manner on every patient requiring it.

To address the challenges presented within healthcare regarding ECGs, innovations in sensor technologies have made it possible to record the electric impulses from the heart in the absence of a conventional ECG setting. The availability of a portable, home-based ECG is a significant medical innovation that can potentially transform medical care, especially due to the ability to immediately transmit the obtained waveforms for expert interpretation and diagnosis [8]. There are several advantages of devices that can utilize smartphone applications, some of which are already commercially available. They can record a rhythm strip when the patient has symptoms and can serve as a useful tool for the long-term monitoring of the patient. This was first recognized in the consensus document on an ambulatory ECG and external cardiac monitoring/telemetry and in the new cardiac pacing guidelines [9,10].

Numerous studies were conducted during the COVID-19 pandemic, which concluded that smartphone-enabled ECG devices are ideal for simple arrhythmia assessments when used in an outpatient setting to identify clinical deterioration in patients with a stable COVID-19 infection, or to preserve hospital capacity needed during the pandemic, and in turn decrease the risk of nosocomial infections, including COVID-19. Nevertheless, doubts still remain whether such ECG devices may be adequate for a more complex ECG evaluation [11].

Currently, several devices on the market may aid in detecting arrhythmias–primarily atrial fibrillation. All available devices are small and lightweight; however, they only record, store, and transmit single-lead ECG recordings. Therefore, these devices only offer limited information compared to a conventional 12-lead ECG. There is one device on the market that enables the reconstruction of a 12-lead ECG; however, this device features a cable that complicates the process of obtaining an ECG, leading to increased time needed to obtain an ECG as well as time in between two ECGs because of the need to disinfect the equipment, thus negatively impacting the usability and feasibility of the device. Up to now, no device on the market can provide a conventional 12-lead ECG recorded by the patient without the use of cables or additional equipment [12,13,14].

The KardioPal is a novel three-lead-based ECG reconstructive technology designed as a handheld device featuring integrated electrodes which can be connected to a smartphone via a Bluetooth link. The device is small and lightweight, having two finger electrodes and two chest electrodes for ECG signal acquisition. The whole process of setting up the device and obtaining a recording takes approximately 60 s without the additional use of cables and sticky electrode patches. The obtained recordings are 30 s in duration with a 500 Hz sampling rate. The KardioPal is simple to use and the accompanying smartphone application provides practical step-by-step voice instructions, allowing patients to successfully use the device without the help of a healthcare professional. A three-lead-based KardioPal reconstruction of a 12-lead ECG is based on the proprietary algorithm presented and clinically validated in the previous publications [15,16,17,18,19]. Briefly, we had initially demonstrated that the standard 12-lead ECG could be numerically reconstructed with high accuracy on the base of the patient transformation matrix previously calculated in the calibration process [19].

**Figure 1:**
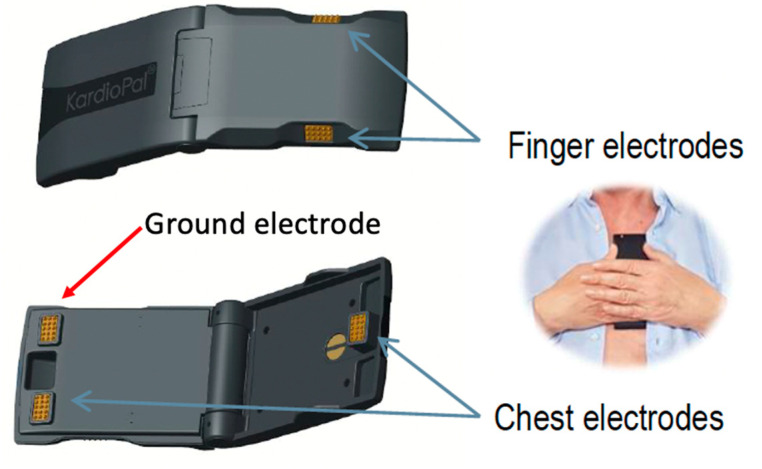
KardioPal device front and back in the unfolded measuring position (**left**) and the correct position of the KardioPal device during the ECG recording (**right**). The vertical distance between the Ag/AgCl plated chest electrodes measures 130 mm when unfolded.

The calibration device is a 15-lead ECG, simultaneous recording of the standard 12-lead ECG and the addition of three special ECG leads (V_b1_, V_b2_, V_b3_) that are specific to the KardioPal technology. This uses two finger electrodes and two chest electrodes (Figure 1). The potential difference between the left finger and two chest electrodes is measured with respect to the right finger electrode as a reference point. The potential difference between the finger electrodes corresponds to Lead I of the 12-lead standard ECG. The positions of the chest electrodes are chosen specifically to compose, together with the finger electrodes, a lead system that is as close to orthogonal as possible. Using the information obtained from the three special leads, the reconstruction algorithm calculates an individual transformation matrix for every patient. This patient-specific matrix enables the reconstruction of a 12-lead ECG from the patient’s information transmitted from the KardioPal device using a simple matrix multiplication of the transformation matrix.

V_12L_ = **T** × V_KP_

where the conventional 12 ECG leads are represented with the vector:

V_12L_ = (I, II, III, aVR, aVL, aVF, V_1_, V_2_, V_3_, V_4_, V_5_, V_6_)

While the three special leads transmitted from the mobile KardioPal device are with the vector:

V_KP_ = (V_b1_, V_b2_, V_b3_)

The transformation matrices (**T**) are patient-specific and were computed using the least-square error method during the calibration procedure. 

Initially, the KardioPal was intended to be used as a telemetric system consisting of: (1) a three-lead ECG device for self-application in environments outside the hospital, (2) a smartphone for the signal recording control and transmission to the diagnostic center, and (3) software for the reconstruction of the standard 12-lead ECG. As described, it was designed to use the patient-specific ECG reconstruction matrix to synthesize a 12-lead ECG [19]. This ultimately required a calibration procedure; however, in order adapt to the occupational limitations during a pandemic, we opted to eliminate the calibration procedure to minimize patient–operator contact and instead used a population-based matrix.

Instead, the original algorithm using the patient-specific matrix was replaced by a unique, population-based matrix applicable to all patients. Such a population matrix reflects the averaged signal characteristics of patients recorded in the previous calibration studies. For that purpose, the population matrix used for KardioPal reconstruction in this study was derived from the pool of all patients participating in the previously published studies using this reconstructive technology [15,16,17,18,19], including overall 285 patients with 285 ECG pairs.

The primary aim of the study was to examine the accuracy of clinically relevant reconstruction matching off an ECG obtained by the KardioPal technology compared to the 12-lead ECG as the gold standard. For that purpose, matching between the two ECG techniques was assessed by consensus of expert cardiologists using a modified 3-point Likert scale (adequate, acceptable, and inadequate). Clinically relevant ECG matching was considered achieved if the matching was assessed as adequate or acceptable.

The secondary aim was to assess the strength of the relationship of distinctive ECG parameters (P wave, PQ interval, QRS complex, QT interval, and T wave duration, and the amplitude, polarity, and axis of the P wave, QRS complex, and T wave) between the KardioPal reconstructed and standard 12-lead ECG recordings.

Additionally, the study aimed to evaluate the feasibility and time required to obtain a KardioPal reconstructed 12-lead ECG recording in the conditions imposed by the COVID-19 pandemic.

## 2. Materials and Methods

### 2.1. Study Design and Population

The prospective, nonrandomized, single-center, adjudicator-blinded trial included 102 consecutive patients who were 18 years or older, from October 2020 to November 2020, who presented to the Institute for Cardiovascular Diseases “Dedinje” in Belgrade, Serbia, on an ambulatory visit or were admitted to the cardiology department. Exclusion criteria were: age under 18, inability or unwillingness to sign the informed consent form, and presence of mental or motor impairments which limited cooperation or ability to obtain an ECG recording. All patients were given an informed consent form to sign and were informed about their right to withdraw for any reason and at any stage of the study. The study was approved by the institutional ethics committee.

### 2.2. Data Collection

Baseline patient characteristics, including age, gender, body mass index, medical history, risk factors, and medications, were collected for all patients. A standard 12-lead ECG was recorded using the General Electric MAC 800 with a standard paper speed of 25 mm/s and amplitude of 1 mV/10 mm. The filter parameters of the standard ECG recorder were set to a 0.05 Hz cutoff for low-frequency filtering, and a 150 Hz cutoff for high-frequency. All ECG recordings were stored in both paper and digital forms as PDF and xml formats, respectively. After the standard ECG was obtained, a KardioPal recording was performed within the following 15 min. 

To obtain the KardioPal recording, the physician would pass the device to either the patient’s hand or place it directly onto the patient’s chest while the patient’s head was turned to the opposite side of the physician. The patient was instructed to place their thumbs on the finger electrodes located on the sides and follow the voice instructions from the smartphone application. For the device to be in the proper position, the longest edge of the KardioPal must coincide with the left edge of the sternum, while the upper edge of the KardioPal device is positioned approximately three fingers below the clavicle, as shown in Figure 1. The KardioPal recording was performed in a supine position for the in-patient study population and in a sitting position with a slightly reclined backrest for the out-patient study population. All recorded KardioPal ECG data were transferred automatically to the smartphone via Bluetooth using the KardioPal application. The application provides feedback to the patient concerning the proper device positioning and the quality of the obtained signal. Recorded data were sent directly to the telemonitoring center for 12-lead reconstruction and further analysis.

### 2.3. Data Analysis

The diagnostic accuracy of the KardioPal device under the pandemic conditions was assessed by comparing the reconstructed 12-lead ECG with the findings of a 12-lead digital ECG recording obtained by standard ECG technology as a gold standard. The assessment of respective ECG pairs was performed by a team of three independent cardiologists. They blindly compared the respective ECG pairs without knowledge of the recording technology or other patients’ clinical and demographic characteristics. The final decision on the matching of each ECG pair was made with the consensus of all evaluators at a joint meeting.

The clinically relevant matching between the two ECG techniques was assessed by a modified 3-point Likert scale (adequate, acceptable, and inadequate) using the criteria defined in Table 1.

The clinically relevant ECG reconstruction matching was considered reached if the final assessment was an adequate or acceptable reconstruction (3 or 2 points).

### 2.4. ECG Signal Parameters Matching Analysis

**Figure 2:**
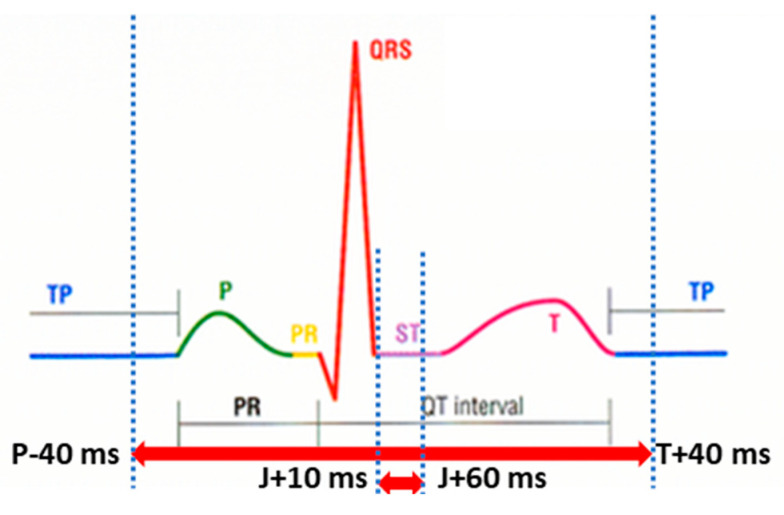
Intervals used in signal comparison: whole heart cycle (P-40 msec to T wave end + 40 msec) and ST segment (ST segment is defined as the interval from J + 10 msec to J + 60 msec).

The numerical matching analysis of the distinctive ECG signal parameters included measuring the duration of the: P wave, PQ interval, QRS complex, QT interval, and T wave, as well as the amplitude, polarity, and axis of the P wave, QRS complex, and T wave. Durations were recorded in milliseconds (msec), amplitudes were recorded in millivolts (mV), and polarities were recorded as positive (+) or negative (−). Amplitude values (voltages) were determined at the point of the maximum amplitude of the QRS complex, T wave, and P wave, as well as a mean amplitude on the ST segment (the ST segment is defined as an interval from J + 10 msec to J + 60 msec). Maximum amplitude implies either negative or positive values (Figure 2).

### 2.5. Efficiency Analysis

For the purpose of the study, efficiency was defined as the time needed to successfully perform the ECG recording. The KardioPal recording time was defined as the time from the start of the smartphone application with the simultaneous positioning of the device on the patient’s chest to the moment when the 12-lead reconstructed ECG recording was available to the physician on the screen of a tablet or smartphone. The standard ECG recording time was defined as the time from placing the first electrode to obtaining the paper ECG record. 

### 2.6. Statistical Analysis

Descriptive statistics were performed, and all results are presented as count (%) and mean ± standard deviation (SD) or median with interquartile range (IQR). Categorial variables were compared using the chi-squared test, while continuous variables were compared using parametric (one-way ANOVA) or nonparametric (Mann–Whitney U test) depending on the normality of the distribution. The strength of the linear relationship between distinctive ECG signal parameters of a reconstructed and standard 12-lead ECG recordings was assessed by the Pearson correlation test.

Statistical analysis was performed using SPSS 29.0 (IBM Corp. Released 2022. IBM SPSS Statistics for Windows, Version 29.0. Armonk, NY, USA: IBM Corp.). A *p*-value less than 0.05 was considered statistically significant. 

In addition, the diagnostic value of the KardioPal recording compared to the standard 12-lead ECG was assessed using the standard methods to calculate sensitivity, specificity, predictive value, and diagnostic accuracy of medical tests; all showed the corresponding 95% confidence intervals (95% CI).

## 3. Results

### 3.1. Demographic and Clinical Characteristics

The study was initially conducted on 110 patients, of whom 8 were excluded. Seven patients were excluded due to an incomplete 12-lead recording, while one patient was excluded due to an inability to record a technically correct KardioPal signal. After repeated attempts, a high level of noise could not be alleviated due to severe trembling of the patient’s hands (probable diagnosis of essential tremor). For the analysis, 102 pairs of technically correct ECG recordings (standard 12-lead ECG and three-lead KardioPal recordings) were used. The total feasibility of obtaining a KardioPal recording was 102/103 (99%). The majority of subjects enrolled in the study were men (60.8%) with an average age of 57.0 ± 14.3 years. The demographic and clinical characteristics of patients who participated in the study are shown in Table 2. The study population can be considered representative of everyday practice given the high percentage of patients older than 65 years (24.5%), the high percentage of obese patients (23.5%), as well as the representation of women (39.2%).

### 3.2. ECG Diagnostic Findings

The significant ECG abnormalities on a standard 12-lead ECG are shown in Table 3. These findings were confirmed by the consensus of experts. Seven ECGs had multiple abnormalities. From the group of patients presenting with ST elevation, the diagnosis of STEMI was confirmed by elevated values of cardiac enzymes as well as by coronary angiography.

Variables are presented as N (%). ECG-electrocardiogram; AV—atrioventricular; RBBB—Right Bundle Branch Block; LBBB—Left Bundle Branch Block; IVCD—Intraventricular Conduction Delay; LVH—Left ventricular hypertrophy; WPW—Wolff–Parkinson–White (WPW); VPCs—Ventricular premature complexes.

### 3.3. ECG Clinical Matching

**Figure 3:**
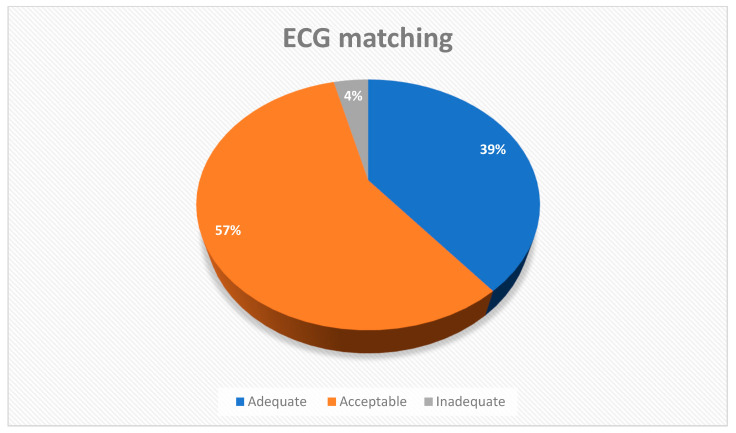
Appropriateness of ECG clinical matching between KardioPal reconstructed and standard 12-lead ECG records.

The results on the appropriateness of the ECG clinical matching, according to the criteria set in Table 1, performed by consensus of three expert cardiologists, are shown in Figure 3.

### 3.4. Diagnostic Accuracy of Clinically Relevant ECG Reconstruction Matching Obtained by the KardioPal Technology

In 4 of the 102 ECG recordings we found the matching to be inadequate, while of the remaining 98 ECG recordings 40 recordings had adequate and 58 acceptable matchings. Out of four inadequate KardioPal recordings, two were false-negatives, and two were false-positives. A closer analysis of the two false-negative ECG recordings, being a real shortcoming with potentially significant clinical consequences, reveals that an inferior scar was neglected in the first case; in the second case, an existing ST segment and T wave changes were neglected. 

Further analysis of the second case, the two false-positive ECG recordings, showed that the patient had intermittent ST changes that day. Since the KardioPal ECG and standard 12-lead ECG were not recorded simultaneously, it is possible that, at the time of recording, the changes did not exist. 

Our results confirmed the high diagnostic accuracy of clinically relevant ECG reconstruction matching (compared to the 12-lead standard ECG) obtained by the KardioPal technology, with a sensitivity of 96.3%, specificity of 95.8%, and diagnostic accuracy of 96.08%. Diagnostic parameters, including corresponding 95% CI, are presented in Table 4.

### 3.5. ECG Signal Parameters Performance Matching

**Figure 4:**
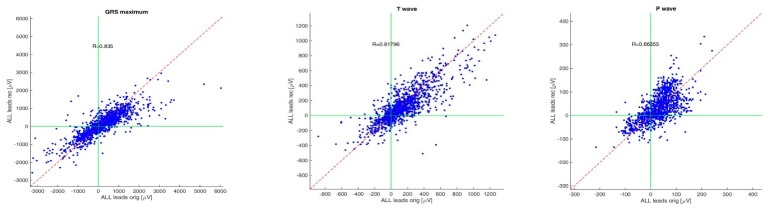
Scatterplots of matching amplitudes at points of maximum amplitude of QRS complex, T wave, and P wave. R-Pearson correlation coefficient.

**Figure 5:**
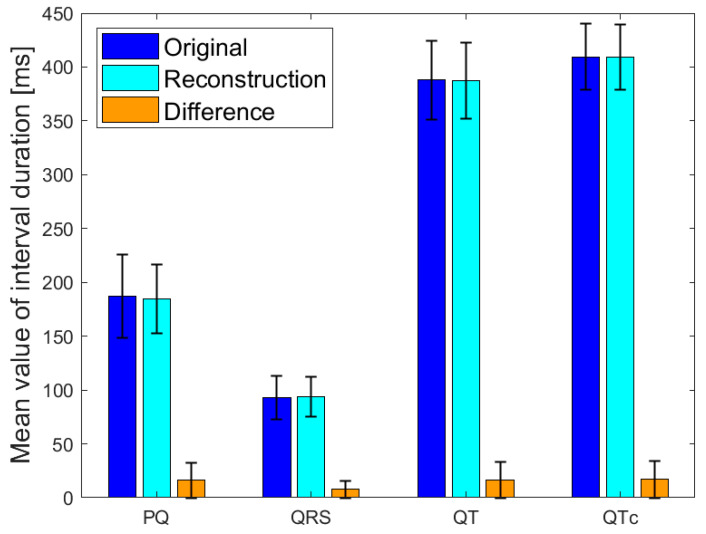
Mean values with standard deviations of PQ, QRS, QT and QTc interval duration.

The correlation between the maximum amplitude of QRS complexes on KardioPal reconstructed and the standard 12-lead ECG recordings is shown in Figure 4. Scatterplots are presented by applying Pearson’s linear correlation test. The correlations of the frontal QRS, T wave, and P wave axes are shown in Table 5. The mean values and standard deviations of distinctive ECG intervals for the original and reconstructed signals are shown in Figure 5.

### 3.6. Efficacy of KardioPal Technology

The mean recording time of the KardioPal ECG was approximately 70 s (69.9 ± 29.5, median time of 61 s). Prolonged acquisition time was only observed in elderly, less cooperative patients and in patients with skin conditions, chest deformities, or pronounced pectoral muscles, and in cases of intermittent internet connection. The mean ECG recording time using a standard 12-lead ECG was approximately 8 min (475.8 ± 144 s, median time of 441 s). Using the KardioPal technology, the recording time was significantly reduced (*p* < 0.05), despite both technologies having exhibited a prolonged acquisition time in elderly patients, less cooperative patients, and patients with skin conditions. Unlike the standard 12-lead ECG, the KardioPal showed an additional limitation among patients presenting with chest deformities, as well as in the case of an intermittent internet connection.

## 4. ECG Clinical Matching Analysis: Case Examples

**Figure 6:**
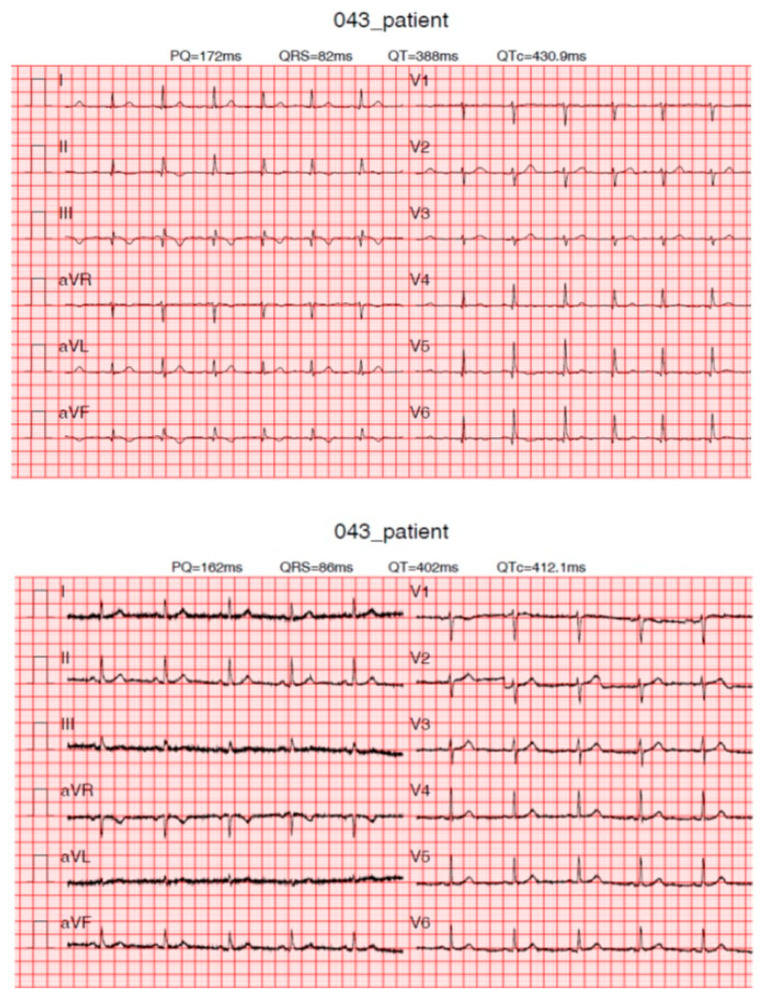
ECGs of patient with inadequate matching. False-positive KardioPal recording indicates an inferior scar (**top**) that is not verified on a standard 12-lead ECG (**bottom**).

Inadequate Matching (Figure 6)

**Figure 7:**
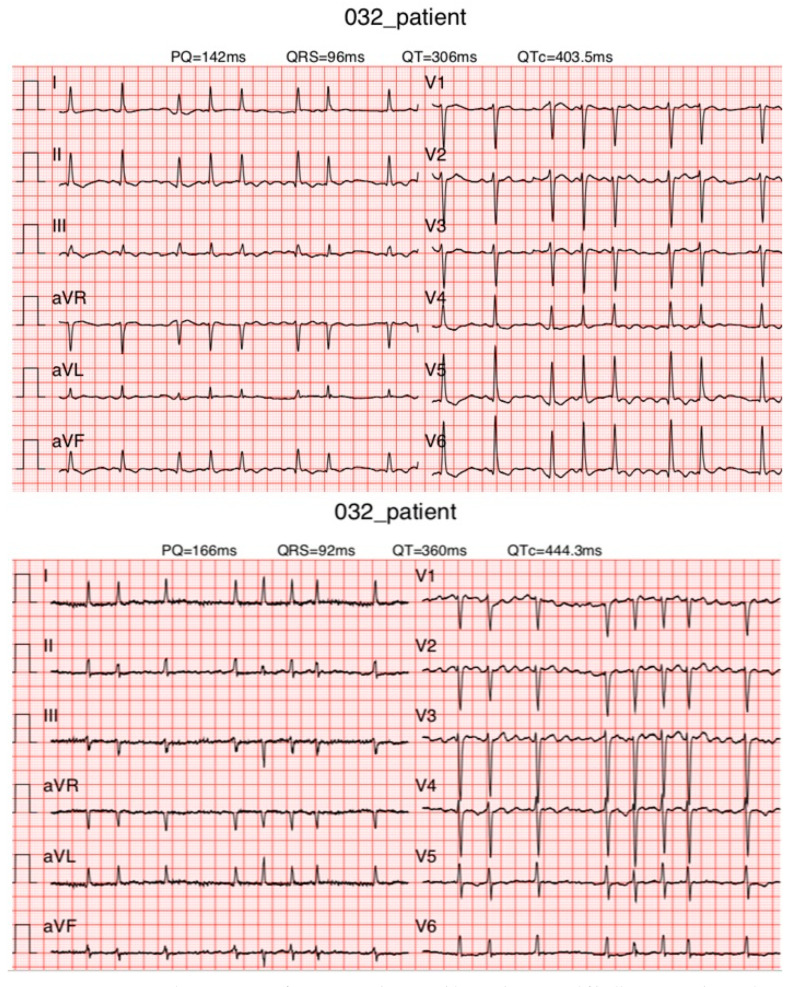
ECGs of a patient with acceptable matching. Atrial fibrillation was observed on KardioPal ECG (**top**) and standard 12-lead ECG (**bottom**).

Acceptable Matching (Figure 7)

**Figure 8:**
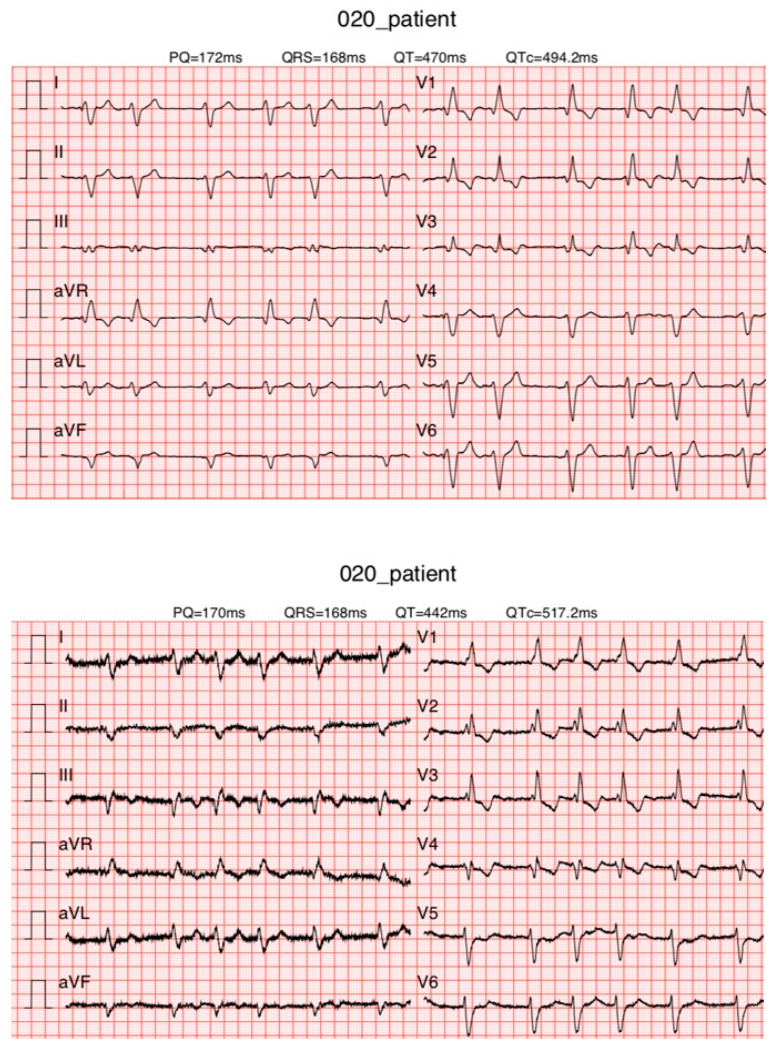
ECGs of a patient with adequate matching. Atrial fibrillation and RBBB were observed on KardioPal ECG (**top**) and standard 12-lead ECG (**bottom**).

Adequate Matching (Figure 8)

## 5. Discussion

The standard 12-lead ECG, obtained from 10 electrodes using an electrocardiograph, is currently accepted as the gold standard for the analysis of ECG changes. In order to adequately perform a standard 12-lead ECG, properly trained staff and a significant amount of space and time are necessary; however, these are not always readily available, as we unfortunately experienced in the healthcare sector during the COVID-19 pandemic. In addition, this technique is not always applicable to critically ill or COVID-19 infected patients. Furthermore, due to the need for assistance to set up the multiple electrodes and cables required to obtain a standard 12-lead ECG recording, the risk of COVID-19 exposure and viral transmission is significantly increased. 

Traditionally, obtaining a 12-lead ECG has been limited to physician offices or hospitals; however, for more than a decade, mobile telemetry devices, with or without a 12-lead ECG reconstruction, have made it possible to obtain an ECG using fewer electrodes without significantly impacting diagnostic accuracy regarding the detection of cardiac arrhythmias [20]. 

Introducing a cableless device capable of simultaneously reconstructing a 12-lead ECG signal using only three electrodes can lead to a revolutionary change in telemedicine as it enables fast and safe acquisition of an ECG without compromising accuracy compared to a standard 12-lead ECG. Our study has shown that a 12-lead ECG can be reconstructed using an average population matrix, as implemented in the modified KardioPal technology presented in this study. Good matching was shown both numerically and clinically (Figure 4, Table 5, and Figure 6, Figure 7 and Figure 8, respectively). This technology has demonstrated high feasibility (99%), specificity (96.3%), sensitivity (95.8%), and diagnostic accuracy (96.1%) to obtain clinically relevant matching of a reconstructed 12-lead compared to the standard 12-lead ECG regarded as the gold standard. Furthermore, this study also showed a significant reduction in the time required to obtain an ECG recording when using the KardioPal compared to a standard 12-lead ECG (69.9 s versus 475.8 s). 

In an upcoming post-COVID study, calibration would be used to create a patient-specific matrix to provide better matching between the reconstructed and standard 12-lead ECG. Previous studies have shown that the best matching could be achieved with an individualized matrix compared to a population-based matrix; however, for the purpose of our study we modified the procedure to eliminate the calibration step to minimize contact due to COVID-19. Our study goes to show that we have 96.3% matching, despite using the population-based matrix. This further supports the theory that such a device that does not require calibration can be useful in telemedicine, particularly in situations with limited access to healthcare.

Regarding feasibility, our study demonstrated that a 12-lead ECG can be reconstructed with the modified KardioPal technology utilizing a population matrix, with high feasibility of 99% (101/102). It is important to emphasize that the high feasibility was achieved in both sitting and supine positions, which was a significant advantage of the KardioPal particularity during the challenging working conditions experienced during the COVID-19 pandemic. 

Several other studies have also demonstrated a high level of feasibility and user-friendliness in emergency and low-resource settings [21,22]. In addition, other researchers also noted that in 10% of patients a standard 12-lead ECG was not adequately performed (due to lead displacement, reversal of the left and the right arm leads, etc.), and that devices with a reduced number of leads have a lower possibility of a technical error during the acquisition stage [23,24]. 

Regarding patients’ characteristics, our results showed a high specificity, sensitivity, and diagnostic accuracy of the KardioPal reconstructed ECG compared to the standard 12-lead ECG in a population with a wide range of baseline ECG changes. This diagnostic performance achieved in our study was greater than that reported by a similarly designed study by Nigolian et al. [25]. The study conducted by McCullough et al. demonstrated that ECG on admission had a significant prognostic implication, especially regarding lethal outcomes, which further highlights the importance of ECG findings in patients presenting with SARS-CoV2. In terms of the structure and content of ECG changes, the ECG findings in their study were comparable to those in our study. This further confirms the applicability of the presented KardioPal technology to aid in diagnosing and monitoring patients presenting with SARS-CoV2 infections [26]. 

The accuracy of the KardioPal technology compared to the standard 12-lead ECG regarding clinical decision-making was assessed by the consensus of expert cardiologists. We demonstrated a satisfactory clinical matching (neither the need for additional diagnostic procedures nor initiation of a different therapeutic approach) between the KardioPal ECG and standard 12-lead ECG in 96% of recordings, of which 39% and 57% of recordings were classified as adequate and acceptable, respectively. The discrepancies which significantly affected clinical decision-making were observed in four (3.9%) recordings. Of the 102 recordings obtained during our study, 2 (1.96%) showed a false-positive, and 2 (1.96%) showed false-negative findings.

In clinical practice, false-positives can lead to additional diagnostic procedures, thus significantly increasing the diagnostic time, but without patient harm. On the other hand, false-negative recordings can harm the patient as they can lead to serious diagnostic and therapeutic errors. Studies have noted a 4% possibility of ECG misinterpretation due to the artifact alone, with an increased possibility of ECG misinterpretation observed in telemonitoring [27]. Regardless of the technology used, it is the physician who interprets the recording and is responsible for validating the final report. Clinical caution is always required, especially when the 12-lead reconstructed ECG findings do not correlate with the patient’s presenting symptoms, clinical findings, or results obtained by other diagnostic tests.

Concerning the choice between the utilization of individual versus population matrix in terms of the overall correlation of reconstructed ECG leads with a standard 12-lead ECG recording, there is a body of evidence showing that synthesizing ECG leads using the individual matrix is superior to using the population matrix [28,29,30,31]. Conversely, most studies with devices utilizing population matrix technology, such as the EASI by Philips, showed good ECG recording matching during myocardial ischemia [32,33,34]. Despite the promising results, the British health authorities issued a warning about the limitations of the technology in patients with chest pain, referring to the observed cases where errors occurred in the reconstruction of the ST segment [35]. However, our study–conducted in a real-life scenario on consecutive patients with different clinical situations–supports the clinical utility of utilizing a population matrix for 12-lead ECG reconstruction.

Furthermore, the study demonstrated excellent comparability of distinctive ECG time intervals, especially the mean values, with a narrow dispersion and minor differences between the reconstructed 12-lead KardioPal ECG values and standard 12-lead ECG. This further confirms the clinical reliability of the KardioPal device, particularly in monitoring PR, QT, and QRS intervals. The possibility of using the KardioPal device to determine and monitor QT and QTc intervals can be of particular significance for individual clinical cases and therapeutic protocols [36].

In addition, the study also demonstrated a sevenfold reduction in time required to obtain an ECG when using the KardioPal device compared to the standard 12-lead ECG (69.9 s versus 475.8 s), further emphasizing a considerable timesaving, especially in cases when the patient requires serial ECG monitoring. 

### Study Limitations

These results represent a single-center experience with novel technology. Two sets of ECGs were neither recorded simultaneously nor with the patient in the same position. Considering the healthcare occupational limitations during the pandemic, simultaneous testing was not practical due to space restrictions, namely the cables of the chest leads could impede the patient’s ability to maintain the proper measuring position with their arms relaxed for the prescribed time to obtain a good signal quality. 

As a result, the standard 12-lead ECG was recorded first, and shortly thereafter, the KardioPal ECG was recorded, having a maximal time difference of 15 min between acquisitions. Despite the narrow time gap between the two ECGs, there is still a possibility of discrepancies in ECG findings, especially in clinically unstable patients such as those with acute or paroxysmal events. While we had an adequate number of subjects to obtain statistically significant results, we did not reach the number of subjects needed for the assessment of predictors of KardioPal ECG diagnostic failures. Furthermore, we measured acquisition time for the KardioPal ECG from the moment of starting the application until the reconstructed ECG recording was available to the physician on the tablet or phone screen, not including the time needed for instructing patients, which was, on average, 30 s. 

Future studies should provide simultaneous ECGs acquisition and include multiple centers with a larger patient population to further evaluate the diagnostic accuracy and assess predictors of diagnostic test failure.

## 6. Conclusions

The novel three-lead-based ECG reconstructive technology by KardioPal is a promising technology to reconstruct a standard 12-lead ECG with high feasibility and diagnostic accuracy. This technology can significantly reduce acquisition time, thus helping to reduce the risk of potential viral transmission. Technologies such as KardioPal have a special importance within the healthcare system and telemedicine as they can significantly reduce the burden on healthcare workers, especially during pandemics. Therefore, the KardioPal device may complement and extend the use of the standard 12-lead ECG during pandemics and upcoming situations of healthcare overload. Compared to a standard 12-lead ECG, regarded as the gold standard, the KardioPal reconstructive technology has shown promising clinical results demonstrating high feasibility, specificity, sensitivity, and diagnosing accuracy; however, additional studies should be performed to assess particular ECG abnormalities with the aim of continuous improvement of current technology. 

## Figures and Tables

**Table 1:** Clinical matching criteria for classifications of ECG readings.

Adequate	3 Points (Pass)	No significant differences in signal shapes or voltages
Acceptable	2 Points (Pass)	Noticeable differences in signal shapes or voltages, but these differences do not affect the appropriate clinical decision-making (no need for additional diagnostic procedures or different therapeutic approach).
Inadequate	1 Point (Fail)	Significant differences in signal shapes or voltages that affect the appropriate clinical decision-making

**Table 2:** Demographic and clinical characteristics.

Age	57.0 ± 14.3
>65 years	25 (24.5%)
BMI	27.4 ± 3.8
>30	24 (23.5%)
Gender (Male)	62 (60.8%)
Risk factors:	
Hypertension	67 (65.6%)
Diabetes mellitus	12 (11.7%)
Dyslipidemia	38 (37.2%)
Heredity	47 (46.1%)
Smoking habit	77 (75.5%)
Previous myocardial infarction	16 (15.8%)
Therapy:	
Beta-blocker	59 (57.8%)
ACE inhibitor	47 (46.1%)
Diuretic	29 (28.4%)
Antiplatelet drug	47 (46.1%)
Anticoagulant drug	19 (18.6%)
Nitroglycerin	7 (6.8%)
Statins	36 (35.2%)

Continuous variables are presented as mean ± standard deviation, categorical variables as N (%). BMI—body mass index, ACE—Angiotensin-converting enzyme.

**Table 3:** ECG findings.

Atrial Fibrillation	14 (13.7%)
First-degree AV block	3 (2.9%)
Inverted T wave	6 (7.8%)
LBBB	4 (3.9%)
RBBB	4 (3.9%)
IVCD	3 (2.9%)
LVH	3 (2.9%)
Pacemaker	1 (1.0%)
Q wave	6 (5.9%)
Sinus Tachycardia	1 (1.0%)
WPW	1 (1.0%)
VPC	1 (1.0%)
ST segment elevation	4 (3.9%)
ST segment depression	9 (9.8%)
Normal findings	48 (47.1%)

**Table 4:** Diagnostic accuracy of clinically relevant ECG reconstruction matching obtained by the KardioPal technology (compared to the 12-lead ECG as a gold standard).

Sensitivity	96.30% (87.25–99.55%)
Specificity	95.83% (85.75–99.49%)
Prevalence of pathological findings	52.94%
Positive predictive value	95.83% (85.50–98.90%)
Negative predictive value	96.30% (86.99–99.02%)
Diagnostic accuracy	96.08% (90.26–98.92%)

Variables are presented as % (95% CI).

**Table 5:** Mean QRS complex, T wave, and P wave axes.

	QRS (S)	QRS (K)	T (S)	T (K)	P (S)	P (K)
Axes (degrees)	19.35	10.87	26.63	21.20	38.84	33.41
Standard deviation (degrees)	36.89	36.61	62.18	65.05	44.62	48.82

QRS (S)—mean QRS axes recording obtained using the standard 12-lead ECG, QRS (K)—mean QRS axes recording obtained using KardioPal, T (S)—mean T wave axes recording obtained using the standard 12-lead ECG, T (K)—mean T wave axes recording obtained using KardioPal, P (S)—mean P wave axes recording obtained using the standard 12-lead ECG, P (K)—mean T wave axes recording obtained using KardioPal.

## Data Availability

Data supporting results are saved at the server of the Dedinje Cardiovascular Institute. Due to patient confidentiality, raw data are not made public. Anonymized data and all ECG recordings are available from the authors upon request.
